# Chlorogenic acid promotes angiogenesis and attenuates apoptosis following cerebral ischaemia-reperfusion injury by regulating the PI3K-Akt signalling

**DOI:** 10.1080/13880209.2022.2110599

**Published:** 2022-08-18

**Authors:** Yong Fan, Yongkun Li, Yongkai Yang, Kunzhe Lin, Qingqiang Lin, Shenghui Luo, Xiaohui Zhou, Qun Lin, Fan Zhang

**Affiliations:** a Central Laboratory, Affiliated Fuzhou First Hospital of Fujian Medical University, Fuzhou, China; b Department of Neurology, Fujian Provincial Hospital, Shengli Clinical Medical College of Fujian Medical University, Fuzhou, China; c Department of Neurosurgery, Affiliated Fuzhou First Hospital of Fujian Medical University, Fuzhou, China; d College of Life Sciences, Fujian Normal University, Fuzhou, China; e Department of Neurology, Affiliated Fuzhou First Hospital of Fujian Medical University, Fuzhou, China; f Department of Anesthesiology, The First Affiliated Hospital of Fujian Medical University, Fuzhou, China; g Department of Neurosurgery, Affiliated Fuzhou Second Hospital of Xiamen University, Fuzhou, China

**Keywords:** Ischaemic stroke, cerebral infarction, neuronal damage

## Abstract

**Context:**

Chlorogenic acid (CGA) has good antioxidant effects, but its explicit mechanism in cerebral ischaemia-reperfusion injury is still uncertain.

**Objective:**

We studied the effect of CGA in human brain microvascular endothelial cells (HBMECs) under OGD/R damage.

**Materials and methods:**

HBMECs in 4 groups were treated with oxygen-glucose deprivation/re-oxygenation (OGD/R) (4** **+ 24 h), normal no CGA treatment and different concentrations (20, 40 or 80 μM) of CGA. Male C57BL/6J mice were classified as sham, middle cerebral artery occlusion (MCAO), and MCAO + CGA (30 mg/kg/day) groups. Mice in the sham group were not subjected to MCAO. Cell viability, apoptosis, angiogenesis and related protein levels were investigated by CCK-8, flow cytometry, TUNEL staining, tube formation and western blot assays. Infarct volume of brain tissues was analyzed by TTC staining.

**Results:**

CGA curbed apoptosis (from 32.87% to 13.12% in flow cytometry; from 34.46% to 17.8% in TUNEL assay) but accelerated cell angiogenesis of HBMECs with OGD/R treatment. Moreover, CGA augmented activation of the PI3K-Akt signalling (p-PI3K/PI3K level, from 0.39 to 0.49; p-Akt/Akt level, from 0.52 to 0.81), and the effect of CGA on apoptosis and angiogenesis was abolished by an inhibitor of PI3K-Akt signalling. Furthermore, CGA attenuated infarct (from 41.26% to 22.21%) and apoptosis and promoted angiogenesis and activation of the PI3K/Akt signalling in MCAO-induced mice.

**Conclusions:**

CGA effectively repressed apoptosis and promoted angiogenesis in OGD/R-treated HBMECs and MCAO-treated mice by modulating PI3K-Akt signalling. Our research provides a theoretical basis for the use of CGA in the treatment of ischaemic stroke.

## Introduction

Ischaemic stroke (IS), the most common type of stroke worldwide, is triggered by a blockage in a blood vessel that reduces blood supply to a special area of the brain (Amarenco et al. 2009; Donkor 2018; Sarfo et al. 2018). The pathological course of IS is associated with a cascade of signals. Depriving brain tissue of oxygen and glucose causes ion pumps and channels to shut down and the body releases an excess of excitatory neurotransmitters, causing neurons to die (Pulsinelli 1992; Paul and Candelario-Jalil 2021). However, it is disappointing that current neuroprotective drugs are not effective in the clinical treatment of IS. Therefore, we need to further study the pathological process of IS, new therapeutic drugs and related mechanisms of action.

Neuroinflammation caused by IS can disrupt the blood–brain barrier (BBB) that is contained in microvascular endothelial cells (Hawkins and Davis 2005). Blood-derived substances can penetrate the broken BBB, and trigger vasogenic oedema, which leads to amplified mortality in patients (Rosenberg and Yang 2007; Khatri et al. 2012). Consequently, in this research, we exploited oxygen-glucose deprivation/reoxygenation (OGD/R) in human brain microvascular endothelial cells (HBMECs) to mimic IS situation *in vitro* (Chen, Sun, et al. 2019).

Chlorogenic acid (CGA) is a phenolic compound mainly existing in coffee, honeysuckle, chrysanthemum, hawthorn, *Eucommia ulmoides* Oliv. (Eucommiaceae) and other Chinese medicinal materials (Upadhyay and Mohan Rao 2013; Naso et al. 2014; Wang et al. 2016). Previous studies have proved that CGA could be anti-inflammatory and antioxidant (Tosovic et al. 2017; Zhang et al. 2018). At the same time, CGA was involved in the treatment of ulcerative colitis (Zhang et al. 2017; Rtibi et al. 2018). Besides, CGA could also take part in regulating cytotoxicity of human oral tumour cells (Jiang et al. 2000). Moreover, CGA participated in modulated HL‑60 cell apoptosis and growth in human acute promyelocytic leukaemia (Liu et al. 2013). Nevertheless, the specific function of CGA in IS and its mechanism are still indistinct, which will be studied in this paper.

The PI3K-Akt signalling pathway is essential to the human body, participating in various important physiological processes, such as angiogenesis, cell cycle, cell growth, apoptosis, glucose metabolism, etc. (Xie et al. 2019). Additionally, PI3K-Akt signalling derived titanium-tempted cell angiogenic stimulus (Martins et al. 2021). Nonetheless, the effect of the PI3K-Akt signalling pathway in the treatment of IS has not been thoroughly studied. Herein, we will explore the link between the effect of CGA and the PI3K-Akt signalling pathway in IS.

Herein, the central objective of this work was tried to analyze whether CGA mediated apoptosis and angiogenesis in HBMECs with OGD/R treatment and mice treated with MCAO, and explored the specific regulatory pathways. This research will provide a new idea for the handling of IS.

## Materials and methods

### Bioinformatics analysis

PharmMapper (http://www.lilab-ecust.cn/pharmmapper/) was used to predict the pharmacologic target of CGA. DisGeNet (http://www.disgenet.org) was performed to predict the target of IS. Gene ontology (GO) analysis or Kyoto Encyclopaedia of Genes and Genomes (KEGG) pathway enrichment analysis were utilized in biological pathways (BP) analysis or gene product metabolic pathway analysis.

### Cells culture and OGD/R treatment

HBMECs were provided by Chuan Qiu Biotechnology (Shanghai, China), and cultivated in the complete medium (Sigma-Aldrich, St. Louis, MO, USA) with 5% CO_2_ and 95% air at 37 °C.

For OGD/R treatment, HBMECs were cultured in glucose and FBS-free endothelial cell medium for 4 h at 37 °C in a hypoxic incubator (5% CO_2_, 94% N_2_ and 1% O_2_). Cells were then reoxygenated in a complete medium (Sigma-Aldrich) under conditions of 95% air and 5% CO_2_ for 24 h at 37 °C. Normal cells were not exposed to OGD/R. For CGA treatment, HBMECs were coped with 20, 40, 80 or 160 μM CGA (purity: 99.55%; MedChemExpress, Monmouth Junction, NJ, USA) for 28 h. CGA was added meanwhile at the start of OGD and maintained in the culture medium throughout the OGD and reoxygenation. For activation and suppression of the PI3K-Akt signalling pathway, HBMECs were exposed to 30 μM 740 Y-P (the activator of PI3K signalling; Sigma-Aldrich) or 25 μM LY294002 (the inhibitor of PI3K signalling; Sigma-Aldrich) for 24 h prior to OGD/R treatment.

### CCK-8 assay

The cell viability was identified by utilizing a CCK-8 kit (Sigma-Aldrich). HBMECs were cultured in 96-well plates (100 µL; 1 × 10^4^ cells/well). After different treatments, cells were exposed to CCK-8 (10 µL; Sigma) for 4 h and the optical density (OD) values were assessed at 450 nm by use of a microplate reader (Biotek, Winooski, VT, USA). Cell viability was expressed as a percentage of OD value in the normal group. The experiments were performed with three biological replicates and three technical replicates.

### Flow cytometry

Cell apoptosis was assessed using an Annexin V-FITC apoptosis detection kit (Thermo Fisher, Waltham, MA, USA) by flow cytometry. In brief, 2 × 10^5^ HBMECs were placed into 6-well plates and then subjected to the indicated treatment. After that, cells were collected and interacted with binding buffer, followed by staining with Annexin V-FITC and propidium iodide (PI). Stained cells were observed using a flow cytometer (Agilent, Hangzhou, China), and the apoptotic rate was expressed as the percentage of cells with Annexin V-FITC positive and PI positive or negative. The experiments were performed with three biological replicates and three technical replicates.

### TUNEL assay

After various treatments, HBMECs were planted into 24-well plates. Then, TUNEL In Situ Cell Death Detection Kit (Sigma-Aldrich) was implemented to measure cell apoptosis intensity. HBMECs were coped with TUNEL (5 µM; Sigma-Aldrich) and the cell nucleus was exposed to DAPI and observed by a fluorescent microscope (200× magnification; Leica, Shanghai, China). The experiments were performed with three biological replicates and three technical replicates.

### Tube formation assay

Matrigel (80 µL/well; 10 mg/mL; Sigma-Aldrich) was precoated in 96-well plates. After 0.5 h, HBMECs cell suspension (200 µL; 1 × 10^4^ cells/well) was seeded into 96-well plates. After for 4 h, the formed tubes were counted through an inverted microscope (40× magnification; Leica). The tube number was computed as the number of branch points that no less than 3 tubes joined. The experiments were performed with three biological replicates and three technical replicates.

### Western blot

The protein contents of vascular endothelial growth factor A (VEGFA), PI3K, p-PI3K, Akt and p-Akt were evaluated by western blot and the detailed protocol as described previously (Song et al. 2019). In brief, 20 µg of protein isolated from cells were subjected to sodium dodecyl sulphate polyacrylamide gel electrophoresis (SDS-PAGE) and membrane transfer, followed by blocking in 5% non-fat milk. After that, the membranes were incubated with primary antibodies at 4 °C overnight, and then incubated with horseradish peroxidase (HRP)-conjugated IgG (ab205718; 1:10,000; Abcam, Cambridge, MA, USA) for 2 h at room temperature. The antibodies were as follows: anti-VEGFA (ab214424; 1:1000; Abcam), anti-cleaved caspase-3 (ab184787; 1:1000, Abcam), anti-PI3K (ab154598; 1:1000; Abcam), anti-p-PI3K (#4228; 1:600; Cell Signalling Technology, Boston, MA, USA), anti-Akt (ab8805; 1:1000; Abcam), anti-p-Akt (ab18206; 1:1000; Abcam) and anti-β-actin (ab8226; 1:1000; Abcam). Enhanced chemiluminescence reagent (ECL; Sigma) was applied to detect the protein bands. For *in vitro* cell samples, tests were performed with three biological replicates and three technical replicates. For animal tissue samples, tests were performed with six biological replicates and three technical replicates.

### Animal and middle cerebral artery occlusion (MCAO) model

The 8-week-old male C57BL/6J mice were purchased from Charles River Laboratories (Beijing, China), and divided into three groups (*n* = 6 per group): sham, MCAO and MCAO + CGA groups following 1-week housing in a controlled condition with free access to food and water. The MCAO model was established referring to the previously reported protocols (Ni et al. 2022). In brief, a midline incision was created in the anaesthetized mice by 2% isoflurane. After that, the right common carotid artery and external carotid artery were isolated, and then the internal carotid artery was clamped, followed by inserting a nylon suture to occlude the middle cerebral artery. MCAO was conducted for 2 h, and reperfusion was performed for 72 h. In the sham group, mice suffered from similar surgeries without occlusion of the middle cerebral artery. In MCAO + CGA group, 30 mg/kg CGA were administered orally to mice daily for 3 consecutive days after MCAO. After the MCAO for 3 days, all mice were euthanized by 5% isoflurane and cervical dislocation. The brain tissues were collected for infarct volume analysis using 2-, 3-, 5-triphenyltetrazolium chloride (TTC) staining. The apoptosis- and angiogenesis-related proteins in cerebral cortex samples were detected by western blot analysis. The experiments were approved by the Animal Experimentation Ethics Committee of The Affiliated Fuzhou First Hospital of Fujian Medical University.

### TTC staining

The 2 mm tissue slices were incubated with 2% TTC solution for 30 min and then fixed with 4% paraformaldehyde, followed by photographing. The infarct tissue showed a white colour, which was analyzed by ImageJ software. The experiments were performed with six biological replicates.

### Statistical analysis

All tests in this paper were repeated no less than three times. The statistics were conveyed as mean ± standard deviation (SD). These data were investigated by one-way analysis of variance (ANOVA) and Student’s *t*-test by SPSS 21.0. *p* < 0.05 was considered significant.

## Results

### The pharmacologic network of CGA in ischaemic stroke

**Figure 1.**
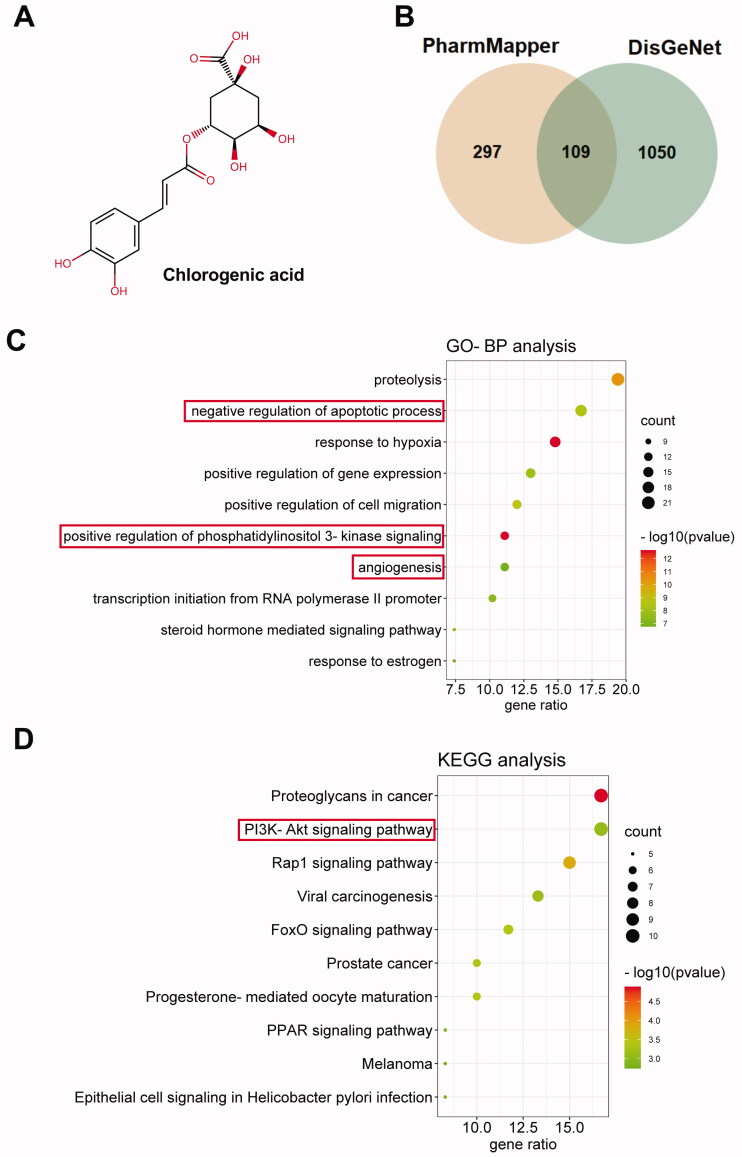
Target prediction and bioinformatics analysis of CGA in ischaemic stroke. (A) The chemical formula of CGA. (B) Venn diagram of shared targets in ischaemic stroke and CGA predicted by using DisGeNet and PharmMapper webservers, respectively. (C) GO-BP analysis of 109 targets and bubble plot for top 10 biological processes using DAVID tool. (D) KEGG analysis of 109 targets and bubble plot for top 10 pathways using DAVID tool.

Firstly, we explored the pharmacologic network of CGA in ischaemic stroke in this part. As displayed in Figure 1(A), the chemical formula is shown. Then, 406 CGA targets were predicted using PharmMapper. At the same time, 1159 targets of IS were predicted by utilizing DisGeNet. Of these predicted targets, 109 overlapped, and further analysis was performed on these targets (Figure 1(B)). Next, these 109 targets were analyzed for GO biological process and KEGG pathway enrichment. Interestingly, these targets were associated with apoptosis, PI3K-Akt signalling and angiogenesis (Figure 1(C)). In addition, these targets were also linked with the PI3K-Akt signalling pathway (Figure 1(D)). Hence, we suggested that CGA might influence apoptosis and angiogenesis in IS by modulating the PI3K-Akt signalling.

### CGA attenuated OGD/R-tempted apoptosis of HBMECs

**Figure 2.**
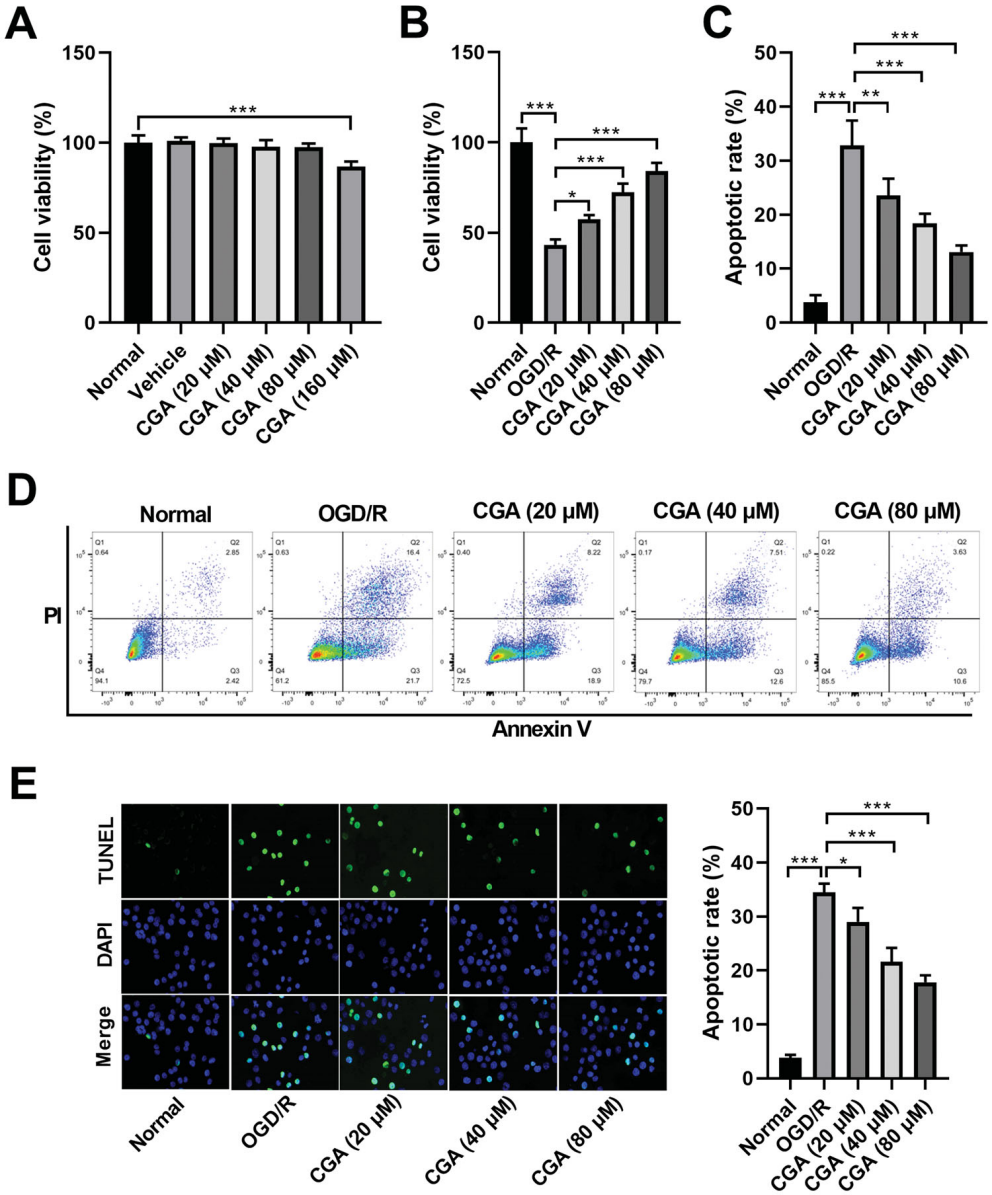
CGA regulated OGD/R-induced apoptosis of HBMECs. (A) The cell viability was evaluated by CCK-8 assay in HBMECs after stimulation with different concentrations of CGA for 28** **h. HBMECs were subjected to OGD/R and CGA treatment. (B) The cell viability was evaluated by CCK-8 assay. (C–E) The apoptosis was investigated by flow cytometry and TUNEL assays. *p** **<** **0.05; **p** **<** **0.01; ***p** **<** **0.001.

We scrutinized the influence of CGA on OGD/R-tempted apoptosis in HBMECs. Primarily, we found that after HBMECs were exposed to a low concentration of CGA (20, 40 or 80 μM), cell viability was not affected (*p* > 0.05). However, the cell viability decreased (86.78%) significantly after CGA treatment with a high concentration (160 μM) (Figure 2(A)). Therefore, 20, 40 and 80 μM CGA were used in the follow-up experiments. In addition, HBMECs viabilities were diminished by OGD/R treatment (43.22%), but this effect was alleviated by CGA co-treatment (57.29%, 72.36%, 84.04%, respectively). Moreover, the higher the concentration of CGA, the better the effect of cell viability recovery (Figure 2(B)). HBMECs apoptosis rate were enhanced after OGD/R treatment (32.87% in Figure 2(C); 34.47% in Figure 2(E)), while this influence was assuaged by CGA co-treatment (23.55%, 18.45%, 13.12%, respectively, in Figure 2(C); 29.01%, 21.59%, 17.8%, respectively, in Figure 2(E)). As the concentration of CGA increased, its inhibition of OGD/R-induced apoptosis became better (Figure 2(C–E)). Therefore, we inferred that CGA could repress the OGD/R-induced apoptosis of HBMECs.

### CGA elevated angiogenesis of HBMECs under OGD/R damage

**Figure 3.**
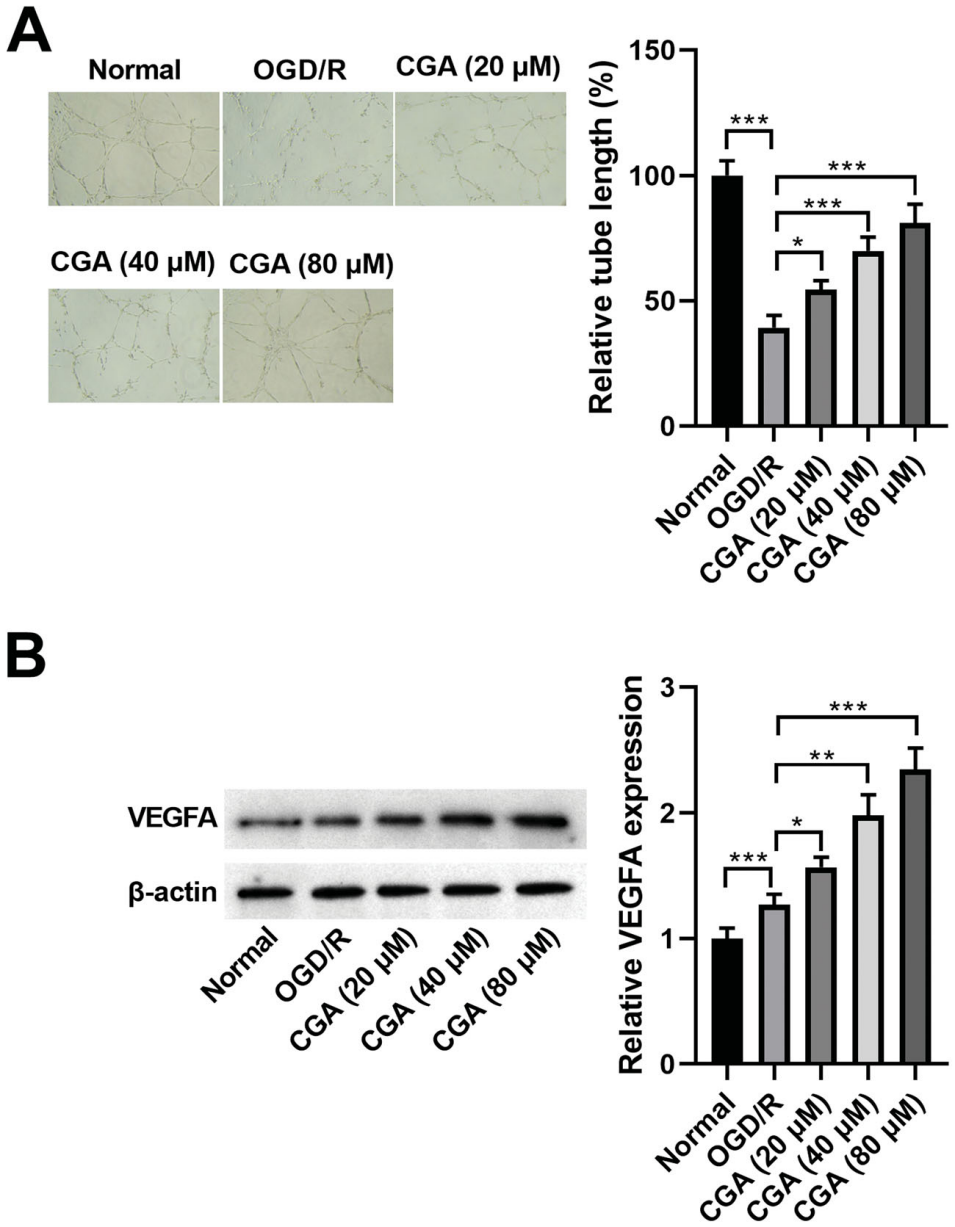
CGA regulated angiogenesis of HBMECs under OGD/R damage. HBMECs were treated with OGD/R and indicated concentrations of CGA. (A) The angiogenesis was estimated by tube formation assay. (B) The VEGFA abundance was tested by western blot. *p** **<** **0.05; **p** **<** **0.01; ***p** **<** **0.001.

Next, we scrutinized the influence of CGA on the angiogenesis of HBMECs with OGD/R injury. After OGD/R treatment, HBMVECs were harvested and then subjected to tube formation assay. After OGD/R treatment, the angiogenesis ability of HBMECs was reduced (39.33%), but this result was lessened by CGA co-treatment (54.39%, 69.92%, 81.10%, respectively). Additionally, the higher the dosage of CGA, the better the influence of angiogenesis ability recovery (Figure 3(A)). In addition, cell angiogenesis is largely dependent on the driving response of VEGFA (Claesson-Welsh and Welsh 2013). Herein, we revealed that the abundance of VEGFA was dwindled by OGD/R treatment (1.26-fold change), whereas enhanced by CGA co-treatment (1.57-, 1.98-, 2.35-fold change, respectively). With the increase in CGA dose, the level of VEGFA gradually augmented (Figure 3(B)). According to these results, we confirmed that CGA stimulated angiogenesis of HBMECs under OGD/R damage.

### CGA promoted activation of the PI3K-Akt signalling in HBMECs under OGD/R damage

**Figure 4.**
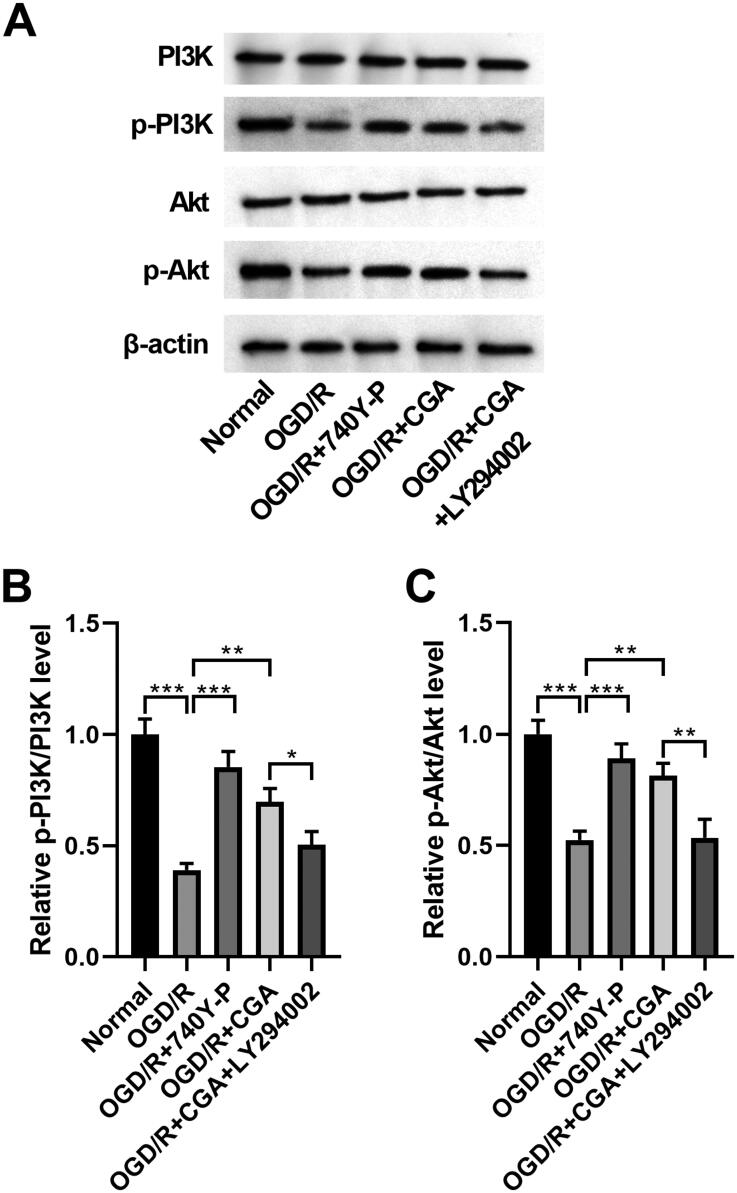
CGA regulated activation of the PI3K-Akt signalling in HBMECs under OGD/R damage. HBMECs were treated with 30** **μM of 740Y-P or 25** **μM of LY294002 for 24** **h prior to treatment with OGD/R and 80** **μM of CGA. (A) The abundance of PI3K, p-PI3K, Akt and p-Akt was detected by western blots. (B, C) The p-PI3K/PI3K and p-Akt/Akt levels were estimated. *p** **<** **0.05; **p** **<** **0.01; ***p** **<** **0.001.

Then, we analyzed the specific signalling pathway by which CGA regulated HBMECs in HBMECs with OGD/R treatment. In this part, we unfolded that the contents of p-PI3K and p-Akt were reduced by OGD/R treatment, whereas this effect was counteracted after 740 Y-P or CGA co-treatment. However, the addition of LY294002 cancelled the recovery effect of CGA on OGD/R treatment (Figure 4(A)). So, we assumed that CGA might have the same effect as 740 Y-P. Besides, the p-PI3K/PI3K and p-Akt/Akt levels declined after OGD/R treatment (0.39-fold change, 0.52-fold change, respectively), but this influence was abolished by 740 Y-P (0.85-fold change, 0.89-fold change, respectively) or CGA co-treatment (0.69-fold change, 0.81-fold change, respectively). Nevertheless, the LY294002 supplement lessened the reverted effect of CGA on OGD/R treatment (0.50-fold change, 0.53-fold change, respectively) (Figure 4(B,C)). We indicated that CGA could contribute to the activation of the PI3K-Akt signalling in HBMECs under OGD/R damage.

### Suppression of the PI3K-Akt signalling via LY294002 reversed the influence of CGA on apoptosis

**Figure 5.**
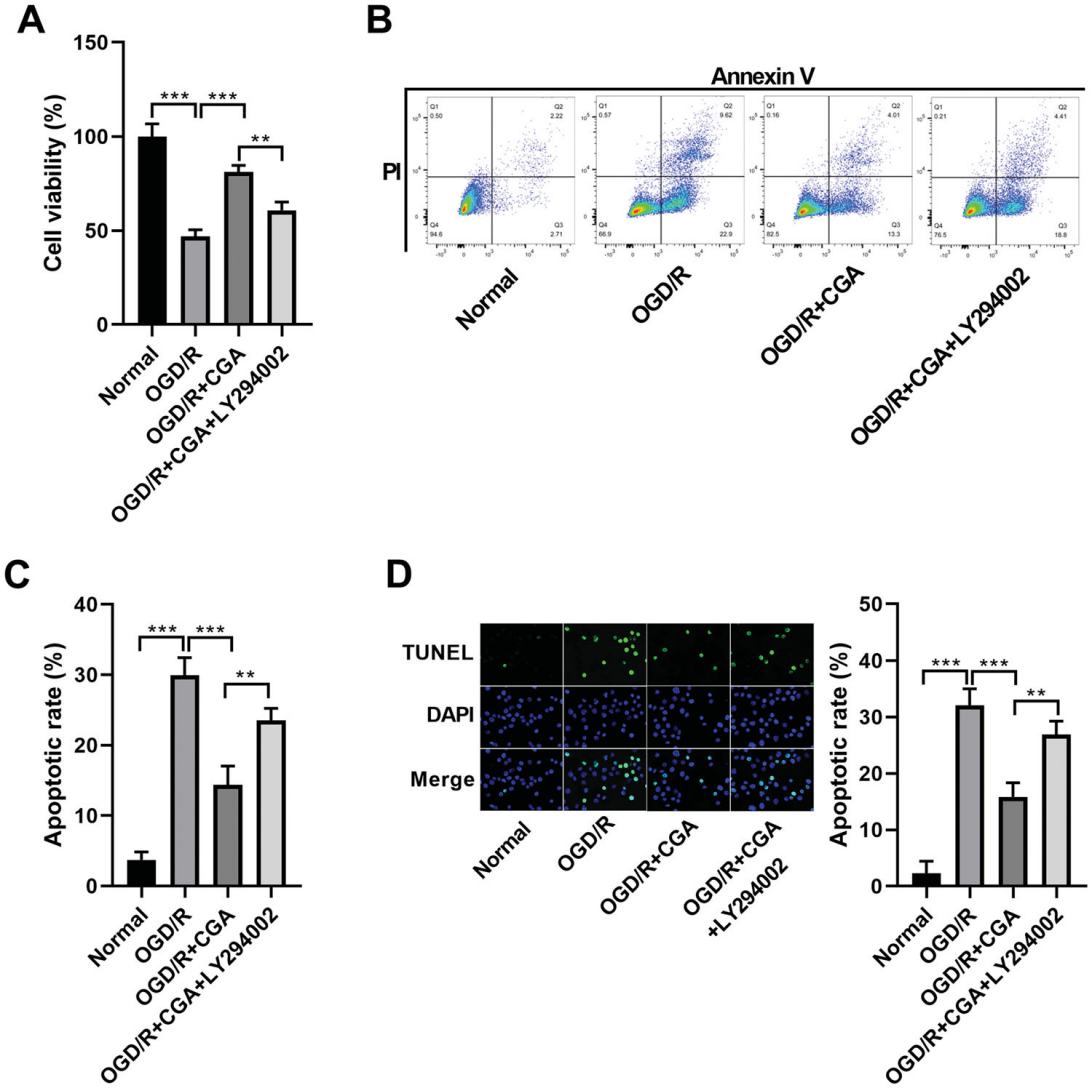
LY294002 regulated the effect of CGA on apoptosis. HBMECs were treated with 25** **μM of LY294002 for 24** **h prior to treatment with OGD/R and 80** **μM of CGA. (A) The cell viability was measured by CCK-8 assay. (B–D) The apoptosis was confirmed by flow cytometry and TUNEL assays. **p** **<** **0.01; ***p** **<** **0.001.

To further verify the relationship between CGA and PI3K-Akt signalling, we carried out a rescue experiment examining cell apoptosis. We confirmed that LY294002 could lessen the promoted effect of CGA on cell viability (from 81.14% to 60.74%) in HBMECs with OGD/R injury (Figure 5(A)). In addition, LY294002 restored the constrained effect of CGA on OGD/R-induced apoptosis (from 14.39% to 23.52% or from 15.85% to 26.89%, respectively) in HBMECs (Figure 5(B–D)). Consequently, we concluded that downregulation of PI3K-Akt signalling reversed the consequence of CGA on OGD/R-induced apoptosis in HBMECs.

### Suppression of the PI3K-Akt signalling through LY294002 reversed the influence of CGA on angiogenesis

**Figure 6.**
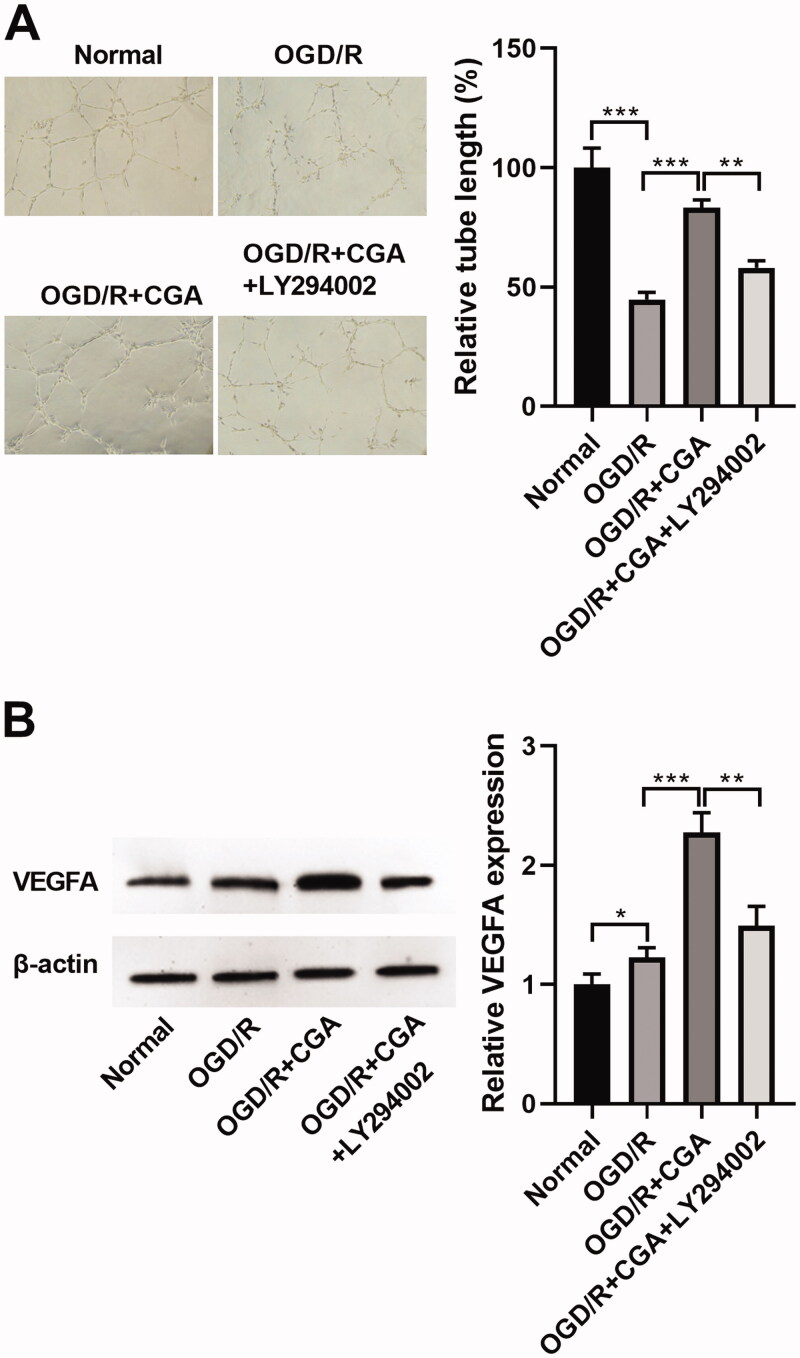
LY294002 reversed the effect of CGA on angiogenesis. HBMECs were stimulated with 25** **μM of LY294002 for 24** **h before treatment with OGD/R and 80** **μM of CGA. (A) Angiogenesis was quantified by tube formation assay. (B) The VEGFA content was detected by western blots. *p** **<** **0.05; **p** **<** **0.01; ***p** **<** **0.001.

In order to prove the link between CGA and PI3K-Akt signalling, we implemented the recover test of cell angiogenesis ability. Interestingly, LY294002 co-treatment reduced the facilitated effect of CGA on cell angiogenesis ability (from 83.30% to 57.95%) (Figure 6(A)) and VEGFA level (from 2.27- to 1.49-fold change) (Figure 6(B)) in HBMECs with OGD/R treatment. Hence, we determined that downregulation of the PI3K-Akt signalling reversed the outcome of CGA on cell angiogenesis viability in HBMECs with OGD/R treatment.

### CGA attenuates neuronal injury in mice after MCAO

**Figure 7.**
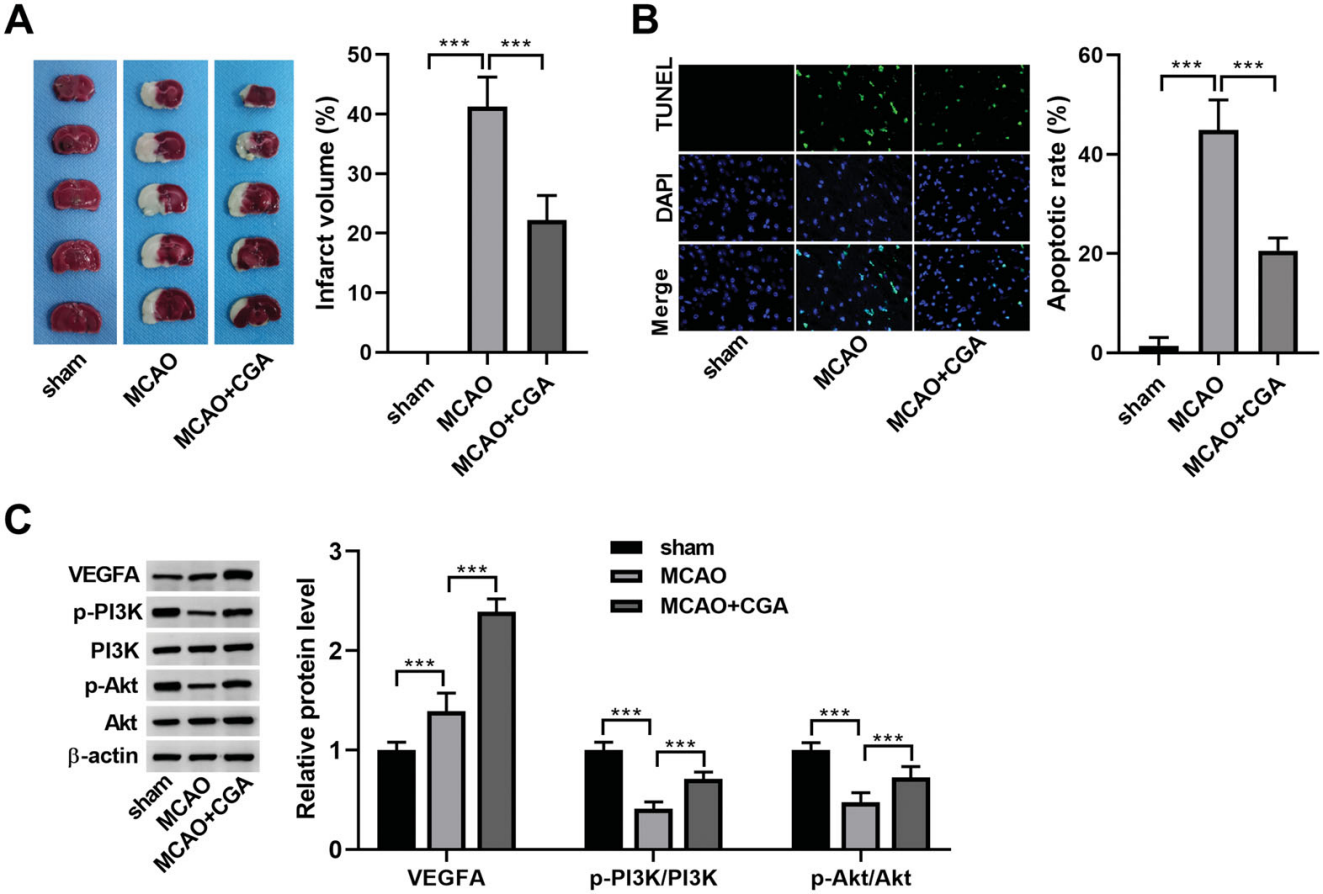
CGA regulated neuronal damage in IS mice induced by MCAO. IS model was established in C57BL/6J mice by MCAO, and then treated with CGA. The mice were divided into sham, MCAO and MCAO + CGA group (*n*** **=** **6). (A) Infarct volume of brain tissues was analysed by TTC staining. (B) Neuronal apoptosis in cerebral cortex of MCAO mice was assessed by TUNEL assay. (C) Protein levels of cl-caspase-3, VEGFA, p-PI3K, PI3K, p-Akt and Akt in cerebral cortex samples were detected by western blot. ***p** **<** **0.001.

To further prove the role of CGA in IS *in vivo*, we established the MCAO-induced IS model. By TTC staining, the infarct volume of brain tissues was markedly increased after MCAO (41.26%), and it was reduced (22.21%) due to CGA treatment (Figure 7(A)). Results of a TUNEL assay showed that CGA treatment attenuated neuronal apoptosis in the cerebral cortex of MCAO mice (Figure 7(B)). Moreover, the apoptosis- and angiogenesis-related proteins and proteins involved in the PI3K-Akt signalling were detected in cerebral cortex samples. As presented in Figure 7(C), the active caspase-3 (cl-caspase-3) and VEGFA levels were obviously increased (3.29- or 1.39-fold change, respectively), while p-PI3K/PI3K and p-Akt/Akt levels were decreased (0.39- or 0.41-fold change, respectively) in cerebral cortex samples in MCAO group compared with the sham group, while CGA treatment increased the levels of VEGFA, p-PI3K/PI3K and p-Akt/Akt (2.39-, 0.71- or 0.74-fold change, respectively), but decreased cl-caspase-3 level (1.57-fold-change). Hence, we concluded that CGA attenuated MCAO-induced apoptosis, and promoted angiogenesis and activation of the PI3K-Akt pathway.

## Discussion

IS can cause a range of serious damage to the body, such as brain cell death caused by oxidative stress, inflammation of the nerve, immune imbalance, etc. (Tao et al. 2020). Among the current treatment methods for IS, recombinant tissue plasminogen activator (tPA) is the only effective drug that has obtained United States Food and Drug Administration (US FDA) certification (Tao et al. 2020). Unfortunately, the onset of IS is rapid and the optimal window for treatment is extremely short, with only 10% of patients being treated effectively (Bansal et al. 2013; Bhaskar et al. 2018). Therefore, we are urgently looking for more treatments and drugs to treat IS. In this paper, we explored the effect of CGA in HBMECs under OGD/R damage.

Many pieces of evidence suggested that CGA had multiple physiological functions. For example, CGA had free radical scavenging activities and had neuroprotective effects under the stimulation of various cell death inducers (Taram et al. 2016). In addition, Lapchak proved that CGA could enhance the therapeutic effect of tPA after multiple infarct IS in rabbits (Lapchak 2007). This indicated that CGA was beneficial for the treatment of IS. Moreover, CGA reduced neuroinflammation in MPTP-poisoned mice (Singh et al. 2018). Besides, Liu et al. (2020) confirmed that CGA could have a neuroprotective influence on the cerebral ischaemia-reperfusion rats via modulating the Nrf2 pathway. Additionally, CGA had many health benefits. Wu et al. (2014) indicated that CGA is effective in the prevention of atherosclerosis and it promotes cholesterol efflux of macrophages in mice. CGA also inhibited platelet activity and thus played an anti-thrombotic role (Fuentes et al. 2014). Lin et al. (2017) revealed that CGA repressed vascular endothelial growth factor (VEGF)-tempted cell angiogenesis ability in HUVECs. Cell apoptosis and angiogenesis are associated with vessel remodelling, and angiogenesis could negatively regulate cell apoptosis (Folkman 2003; Watson et al. 2017). In this paper, according to the results bioinformatics analysis, we deduced that CGA might regulate cell apoptosis and angiogenesis in ischaemic stroke through PI3K-Akt signalling. Additionally, CGA restricted apoptosis but enhanced cell angiogenesis of HBMECs with OGD/R treatment, which was similar to the result of Lin et al. (2017). Our study also confirmed the negative correlation between apoptosis and angiogenesis in ischaemic stroke. However, a previous study reported the anti-angiogenesis role of CGA in lung cancer cells (Park et al. 2015). Angiogenesis is associated with the delivery of oxygen and nutrients to tumour cells, while it may also occur as an advantageous defense response against hypoxia in IS by improving blood supply to the brain tissue (Hong et al. 2019). Hence, the therapeutic roles of CGA might be associated with anti-angiogenesis therapy in tumours and pro-angiogenesis therapy in IS. We hypothesized the dual effects of CGA on angiogenesis in IS and tumour cells might result from different mechanisms through various targets.

According to the results of Chen et al. (2020), the PI3K-Akt signalling pathway was linked with the neuroprotective of transient IS. Moreover, Lin et al. (2017) also illustrated that the anti-angiogenesis action of CGA was linked with phosphorylation of Akt. In addition, resveratrol could offer neuroprotection in stroke by modulating the PI3K-Akt signalling pathway in mice (Hou et al. 2018). Further, ginsenoside Rg1 contributed to cerebral angiogenesis in ischaemic mice by regulating the PI3K-Akt signalling pathway (Chen, Zhang, et al. 2019). Also, *Panax notoginseng* (Burkill) F.H.Chen ex C.Y.Wu & K.M.Feng (Araliaceae) saponins could regulate the PI3K-Akt signalling pathway to inhibit OGD/R-tempted barrier dysfunction in cerebral microvascular endothelial cells (Hu et al. 2018). In this research, we confirmed that CGA augmented activation of PI3K-Akt signalling in HBMECs under OGD/R damage, which was parallel with the description of the early report (Hu et al. 2018). Additionally, the influence of CGA on apoptosis and angiogenesis was eliminated by the inhibitor of PI3K-Akt signalling in HBMECs with OGD/R treatment. Our conclusion echoed the result of Lin et al. (2017) and Chen, Zhang, et al. (2019). Furthermore, the protective roles of CGA in IS were also confirmed in MCAO-induced mice by decreasing infarct and apoptosis, and increasing angiogenesis. However, there are some limitations in our current research. The interaction between the PI3K-Akt signalling and CGA in animals was not studied. Hence, the effects of PI3K-Akt signalling on the role of CGA in animal models should be investigated in the future. Moreover, the potential targets mediated by CGA to regulate the PI3K-Akt signalling should also be explored in further study.

## Conclusions

CGA suppressed apoptosis and stimulated angiogenesis in OGD/R-stimulated HBMECs and MCAO-induced mice through modulating PI3K-Akt signalling. These results proved that CGA had a positive effect on the cure of human IS, and provided a potential theoretical basis for the development of CGA in the clinical treatment of IS.

## Data Availability

All data generated or analyzed during this study are included in this published article and its additional files.
